# Efficient enumeration-selection computational strategy for adaptive chemistry

**DOI:** 10.1038/s41598-022-17938-x

**Published:** 2022-08-29

**Authors:** Yachong Guo, Marco Werner, Vladimir A. Baulin

**Affiliations:** 1 Kuang Yaming Honors School, Nanjing University, Nanjing, 210023 China; 2 Leibniz-Institut für Polymerforschung Dresden e.V., Hohe Strasse 6, 01069 Dresden, Germany; 3 Departament Química Física i Inorgànica, Universitat Rovira i Virgili, Marcel.lí Domingo s/n, 43007 Tarragona, Spain

**Keywords:** Structural properties, Computational models, Computational platforms and environments, Polymers, Self-assembly

## Abstract

Design problems of finding efficient patterns, adaptation of complex molecules to external environments, affinity of molecules to specific targets, dynamic adaptive behavior of chemical systems, reconstruction of 3D structures from diffraction data are examples of difficult to solve optimal design or inverse search problems. Nature inspires evolution strategies to solve design problems that are based on selection of successful adaptations and heritable traits over generations. To exploit this strategy in the creation of new materials, a concept of adaptive chemistry was proposed to provide a route for synthesis of self-adapting molecules that can fit to their environment. We propose a computational method of an efficient exhaustive search exploiting massive parallelization on modern GPUs, which finds a solution for an inverse problem by solving repetitively a direct problem in the mean field approximation. One example is the search for a composition of a copolymer that allows the polymer to translocate through a lipid membrane at a minimal time. Another example is a search of a copolymer sequence that maximizes the polymer load in the micelle defined by the radial core-shell potentials. The length and the composition of the sequence are adjusted to fit into the restricted environment. Hydrogen bonding is another pathway of adaptation to the environment through reversible links. A linear polymer that interacts with water through hydrogen bonds adjusts the position of hydrogen bonds along the chain as a function of the concentration field around monomers. In the last example, branching of the molecules is adjusted to external fields, providing molecules with annealed topology, that can be flexibly changed by changing external conditions. The method can be generalized and applied to a broad spectrum of design problems in chemistry and physics, where adaptive behavior in multi-parameter space in response to environmental conditions lead to non-trivial patterns or molecule architectures and compositions. It can further be combined with machine learning or other optimization techniques to explore more efficiently the parameter space.

## Introduction

Finding a structure or a pattern among numerous possibilities that fits to a certain environment or performs a given function is an attractive target for predictive theoretical methods leading to profusion of scientific creativity. In general, problems of optimal design of materials that mimic, adapt or possess a controlled affinity to external conditions are examples of inverse problems^1^. Each inverse problem is associated with a direct problem, consisting in repetitively solving or simulating a well-defined model system for a concrete set of parameters and conditions^2^. These optimal design or inverse problems can be rather challenging^2^ and in real situations can be computationally expensive without additional conditions or regularization^3^, or require computationally expensive heuristic optimization methods, *e.g.* genetic algorithms^4^ and neural networks^5,6^.

The examples of such optimal design problems in chemistry and physics include the understanding of adaptiveness or binding affinity in the molecular recognition process within the lock-and-key paradigm^7,8^, which turns out to be very sensitive to molecular parameters^9^; the search for polymer sequence design in protein folding^10^, the interaction potentials for optimal self-assembly^11^, the search for dynamic exchange of components in response to environmental conditions^12^, the prediction of a sequence of cell-penetrating peptides^13^, inverse design problems in nanophotonics^14,15^.

A concept of Dynamic Combinatorial Chemistry (DCC) was introduced^16^ to address reversible connections and conformations of basic elements and chemical components that can rearrange and dynamically adapt to changes in the environment, which contrasts with static libraries of prefabricated molecules and compounds. Reversible and adaptive connections between elements can be grouped in virtual combinatorial libraries, while a search through libraries of connections in a changeable environment is another example of inverse problem that needs an efficient solution. This idea of experimentally creating adaptive molecules was proposed even before computational design algorithms were widely used to discover new materials.

Among computer simulation methods, one of them, the Reverse Monte Carlo (RMC) method^17,18^, produces three-dimensional structure of a molecular system that fits a given experimental data that originate from unknown molecular structure, such as, for example, diffraction and scattering data in the problems of inverse scattering or reconstruction from neutron diffraction patterns^19^. This is achieved by direct Monte Carlo simulation of these data from a structural model, in which parameters are adjusted in order to fit the experimental data. Since it is not practical in many situations to explore all possible combinations of parameters of the molecular structure unless the model is very simple, the resulting structure is not unique or exact and there is no guarantee that it produces the best fit of the data. Ideally, a method that exhaustively explores all parameter space tests all possible combinations would provide a guaranteed best solution.

Finding a solution to an inverse problem by exhaustively solving the direct problem in a complete parameter space resembles a brute-force password screening in computer science. However, in contrast to password generation, direct computer simulations can be very computationally expensive, thus making this approach too slow and almost impractical. Meanwhile, explosive growth^20^ of computer power provided by parallel computation on modern Graphical Processing Units (GPUs) can be game-changing in the field of inverse search problems, including direct sampling of the parameter space, thus providing a basis for adaptive chemistry basis on the level of computer simulations.

Nevertheless, many existing GPU implementations of Molecular Dynamics and Monte Carlo simulations are, in fact, adaptations of sequential codes to parallel environments, that cannot fully benefit from the potential speed-ups on modern parallel GPU architectures counting thousands of cores. Limitations in scalability come from the Amdahl law^21^ stating that even a small fraction of sequential code or unavoidable synchronization between cores would dramatically impact the scale-up. Hence, to get a full advantage of a parallel hardware architectures, conceptually new scientific methods that are fully parallel in nature have to be invented or re-discovered.

The architecture of a new generation of processors that are still to come dictates a completely different design of methods for solving the optimization problems. As an example, parallel architecture and explosive increase of computational power was the reason why neural networks, invented in the 80s were computationally too expensive to solve any real problem at that time and became so popular and efficient in the 00s. Despite being computationally more expensive than other direct methods, they scale well with the number of units and weights in the network permitting a large number of layers unless other stochastic methods, mentioned by the reviewer, that scale combinatorially^22^. This could be a similar trend with exact enumeration for inverse search problems in the near future when the number of parallel units would increase even more significantly, although it may be difficult to imagine it now, taking into consideration all limitations present in the modern parallel architectures such as limited shared memory and bandwidth of communication with the global memory. New methods should not only scale well with the number of units, but they should be perfectly scalable.

In the following, we report a theoretical method of solving optimal design problems using highly parallel technique taking advantage of massive parallelization on GPUs. After introducing the method, we demonstrate its performance on two examples: the search for a copolymer sequence that translocates through a lipid membrane at a minimal time and a copolymer sequence that maximizes the load by a copolymer micelle.

**Figure 1:**
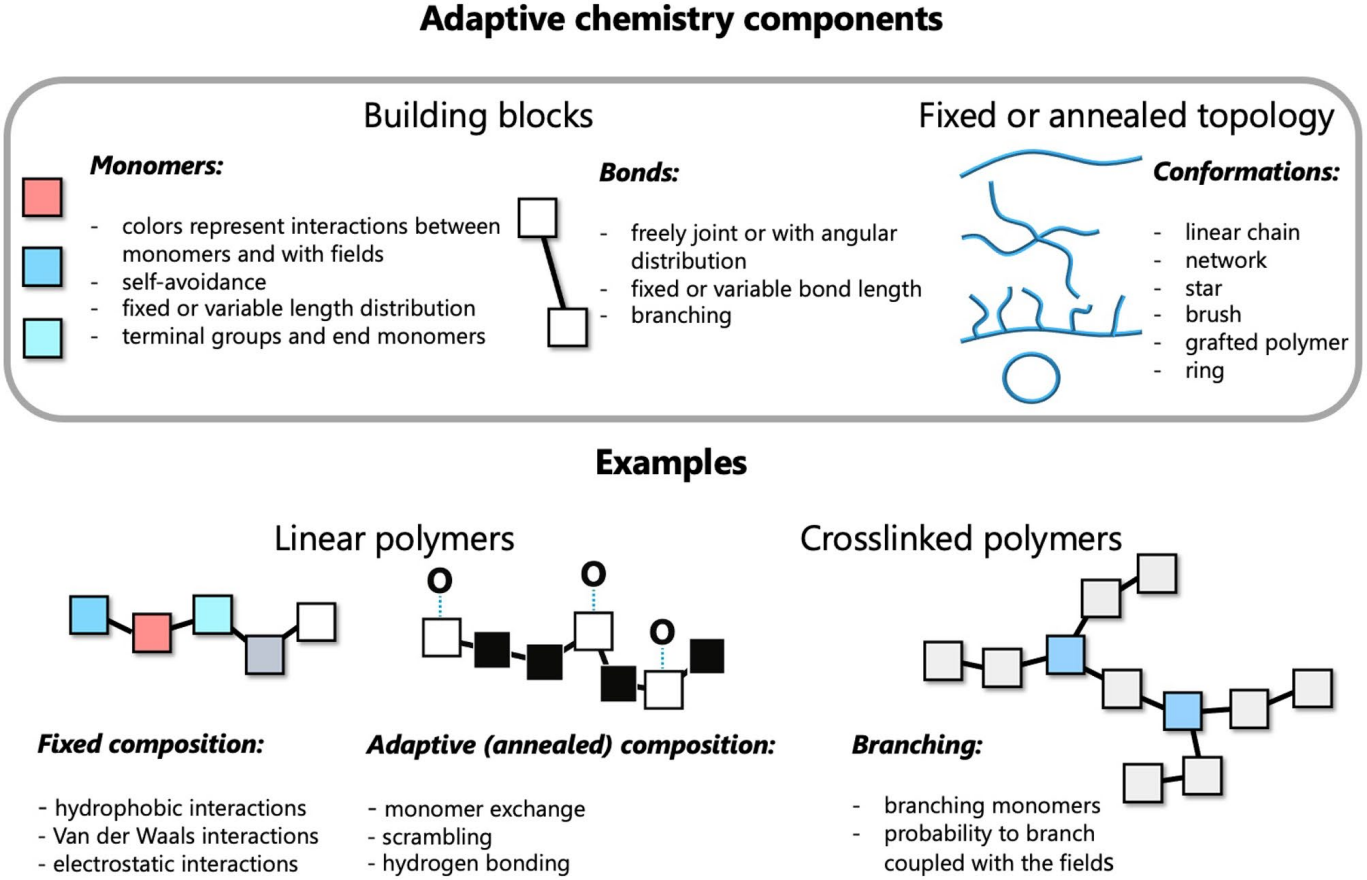
Top: Building blocks and topology of molecules that are created with the enumeration-selection strategy. Conformations are built from monomers connected by bonds and interacting with the fields: concentration fields or external fields. Various types of topology can be addressed. Bottom: three practical examples studied in detail: linear polymer with the composition adjusted to a lipid membrane translocation; linear polymer with hydrogen bonds adapting to the concentration gradient; branched polymer with a field of distributed branching points.

**Figure 2:**
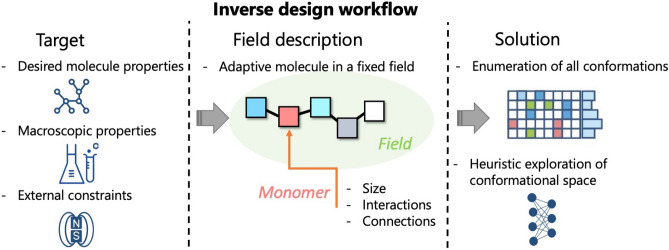
Inverse design workflow.

## Enumeration-selection strategy

Exhaustive search through parameter space implies running massive simulations corresponding to a direct problem in efficient way. This is possible if these simulations are completely independent and do not require communications between the cores and thus would be a subject only to hardware limitations on the number of available cores. For example, such independence of tasks can be realized in the quasi-instantaneous field approximation, where individual molecules interact between each other through fixed or rarely updated mean fields^23^. It was shown that maintaining fields at most recent values and updating them at a certain frequency introduces correlations between the molecules. In such a case, the composition of a molecule, force fields and conformations of a single molecule can be strictly decoupled between each other, thus allowing to generate a molecule composition from building blocks, composition and force fields suitable for massive parallel implementation, Fig. 1. The workflow of the method is depicted in Fig. 2. Once the target parameters are identified: molecule properties and constraints, macroscopic material properties and the restrictions from external fields and conditions; the molecule is digitalized into building blocks (monomers), which are characterized by its size, connections and interactions. This object with annealed structure and parameters is placed in the fixed fields that are dictated by the design target. The fields can be physical fields or chemical gradients/concentrations. Then this adaptive molecule with unknown shape and structure needs to adjust to external conditions and the fields in the inverse enumeration of all conformations search, that can be combined with heuristic methods.

The concept of adaptability implies the external fields as a driving force for the change of the composition or the topology of the adaptive molecules. It can be a concentration fields of monomers, external physical fields, chemical gradients. The building blocks of this method are the monomers that have distinct colors representing interactions with the fields.

This concept is demonstrated in three distinct examples: (i) a linear copolymer of fixed length whose composition is adapted for translocation through a lipid membrane with a minimal time and the composition of a block copolymer in a micelle is optimized for the maximal load; (ii) adaptive linear polymer interacting with the surrounding through reversible links such as hydrogen bonding. The probability of forming a hydrogen bond depends on the concentration field surrounding the monomer; (iii) a branched molecule with annealed topology, where the probability of branching is influenced by the surrounding. Concentration fields of certain components can control the gelification or branching of the molecules, while the concentration fields can be tuned by external environments.

### Basic principles

The method is similar in spirit to Single Chain Mean Field (SCMF) theory^24^ for polymers, where correlations between conformations of a single polymer chain are decoupled and each conformation of the polymer chain is interacting with mean fields independently. Soft matter objects such as micelles and lipid bilayers are, in fact, close to liquids, where molecules move independently and uncorrelated between each over. Thus, in most cases, such an approximation is valid and provides a good results for mechanical properties and self-assembly^25^. Since building blocks in this method are entire molecules with full sampling of conformations, the correlations between monomers inside the molecule are exactly calculated. Density Functional theory (DFT)^26^ has a similar spirit for atoms and small molecules. There are however particular cases, when this approximation is not valid: ion pairs, when two molecules are correlated and move together, movements in gel phase, crystallization, strong electrostatic interactions beyond mean field.

As an example of application of the method, let us consider a linear copolymer chain of a given length *N* and consisting of monomers of types *a*. The monomers of different types differ in interactions between each other and with external fields, which represent the environment. The aim of the method is to find a *sequence* of monomers that satisfy a given design criteria based on the fields, *e.g.* the affinity with the external fields. The sequence consists of monomers from the library, where a color corresponds to a given interactions with concentration and external fields, further denoted by $\Phi$. These monomers are connected between each other by freely joint bonds of fixed length, which can be represented by a cube on a cubic lattice. With such construction, each sequence of monomers can be in many conformational states, described by relative position of monomers in space.

**Figure 3:**
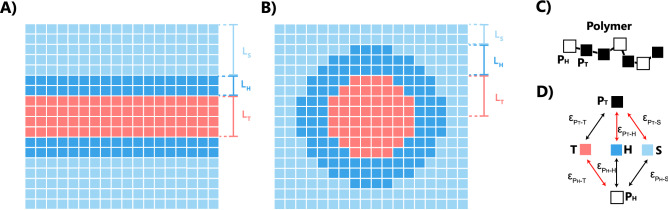
One dimensional fields on a 3D cubic lattice corresponding to (**A**) planar geometry of a lipid membrane, (**B**) spherical geometry of a core-shell micelle. (**C**) Lattice representation of a copolymer sequence: black $P_T$ and white ${P_H}$ cubes represent hydrophobic and hydrophilic blocks, respectively. (**D**) Map of interactions between monomers $P_H$, $P_T$ and fields T, H and S.

We assume that each conformation $\Gamma$ corresponding to a given sequence is independent when placed in fixed fields $\Phi _i(r)$, where *r* is a point in space, Fig. 3. With this, the Hamiltonian of a conformation $\Gamma$ is described by a

1 $$\begin{aligned} \mathscr {H}\left( \Gamma \right) = \mathscr {H}^{intra}\left( \Gamma \right) + \sum _{i}\sum _{a}\int dr U_i^{a}(\Gamma ,r) \Phi _{i}\left( r\right) , \end{aligned}$$

where the integration is over the whole space, $\mathscr {H}^{intra}(\Gamma )$ is the intra-molecular interaction energy resulting from interactions between monomers inside the same molecule; $U_i^{a}(\Gamma ,r)$ is the interaction potential between monomers of type *a* with the field *i* of conformation $\Gamma$ at the position *r*. With this, the total partition function of the system, *Z*, can be written as:

2 $$\begin{aligned} Z\propto\sum _{\Gamma }w_{\Gamma }e^{-\mathscr {H}\left( \Gamma \right) }, \end{aligned}$$

where $w_\Gamma$ are the weights of the conformational distribution, if it was generated with a bias^27,28^. Partition function *Z* provides all thermodynamic properties of the system, while the free energy is given by $\mathscr {F}=-k_BT\ln Z$, where $k_B$ is the Boltzmann constant and *T* is the temperature.

**Figure 4:**
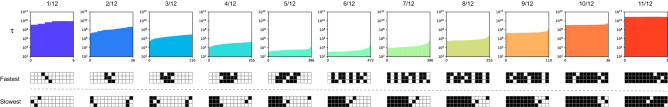
Predicted sequences sorted according to their time of translocation through a lipid bilayer for a copolymer consisting of 12 monomers. Top: Mean escape time $\tau$ as a function of polymer chain sequence number sorted from fastest to slowest. Bottom: the three fastest and three slowest sequences. The color (from blue to red) corresponds to the average hydrophobicity, defined by the fraction of hydrophobic blocks $N_{T}/(N_{T}+N_{H})$ and ranging from 1/12 to 11/12. For comparison, translocation times for homopolymers, 0/12 ($9.90 \times 10^{10}$), 12/12 ($4.96\times 10^{12}$) show two orders of magnitude difference.

Thus, by introducing independent conformations $\Gamma$ placed in fixed fields we provide a basis for a very efficient parallelization scheme, but for the price of neglecting correlations and limiting application to the mean field description. Each conformation $\Gamma$ of a given sequence can be generated independently on a single GPU core with strictly no communication with other cores. This can be realized with an efficient algorithm, such as reported GPU realization of the Rosenbluth algorithm^29^. The weights of the distribution $w_\Gamma$ and the Hamiltonian $H(\Gamma)$ can be calculated on-the-fly without a need to keep them in memory. The resulting $H(\Gamma)$ can be used for classification and ordering of the sequences according to a given design criteria. Thus, such an approach allows for a fast screening of the sequences using full power of parallel architecture of modern GPUs with no limitations of the Amdahl law^21^.

### Realization on a cubic lattice

For numerical purposes, it is convenient to discretize the space and consider sums instead of space integrals in Eq. (1). We assume that the simulation box is divided into three fields as it shown in Fig. 3, hydrophobic field, $\Phi _T$, hydrophilic field, $\Phi _H$ and the solvent, $\Phi _S$. Thus, on a cubic lattice, where each monomer occupies one lattice site, the Hamiltonian, Eq. (1), is written as

3 $$\begin{aligned} \begin{aligned} \mathscr {H}\left( \Gamma \right) =\,&\mathscr {H}^{intra}\left( \Gamma \right) \\&+\varepsilon _{P_{T}-H}\sum _{x,y,z} U^{P_T}_H(\Gamma ,x,y,z) \Phi _{H}\left( x,y,z\right) \\&+\varepsilon _{P_{H}-T}\sum _{x,y,z} U^{P_H}_T(\Gamma ,x,y,z) \Phi _{T}\left( x,y,z\right) \\&+\varepsilon _{P_{T}-S}\sum _{x,y,z} U^{P_T}_S(\Gamma ,x,y,z) \Phi _{S}\left( x,y,z\right) ,\\ \end{aligned} \end{aligned}$$

where $U^{P_T}_H(\Gamma ,x,y,z)$ is the number of contacts of the conformation $\Gamma$ of the $P_T$ polymer at the lattice site (*x*, *y*, *z*), interacting with the field $\Phi _{H}$. Similar definition is applied to $U^{P_H}_T(\Gamma ,x,y,z)$ and $U^{P_T}_S(\Gamma ,x,y,z)$, interacting with $\Phi _{T}$ and $\Phi _{S}$, correspondingly^27,28^. The lattice number (the maximum number of neighbors of a lattice site) is $z=26$ for a cubic lattice. Thus, if one of these sites is occupied by a monomer T or H, or a solvent S, it is considered as a contact with a corresponding energy $\varepsilon$.

The incompressibility condition is imposed by the requirement that each lattice site can only be occupied either by a monomer or by a solvent. The lattice site, occupied by a polymer does not interact with other monomers. We assume that all units, $P_T$, $P_H$, T, H and S, have the same size of one lattice unit and the same interaction range (nearest neighbors only) and differ only in corresponding interaction energies: $\epsilon_{P_T-H}=0.1$ $k_BT$, $\epsilon _{P_H-T}=0.1$ $k_BT$, $\epsilon _{P_T-S}=0.1$ $k_BT$. All other interactions are set to zero (Fig. 3C and D). They are chosen in such a way that if the polymer chain is composed entirely of $P_T$, it would be fully hydrophobic, and if it is composed entirely of $P_H$, it would be fully hydrophilic.

Statistical segments of chain-like molecules are represented as connected unit cubes on a simple cubic lattice. The distance between bonded monomers is constrained to a bond vector set of 26 bond vectors by (1,0,0),(1,1,0),(1,1,1), for each monomer, interacting maximum with the 26 nearest neighbor sites. The excluded volume is implemented via the exclusion of overlap between monomer on the same lattice site, while the distances are expressed in terms of the number of lattice sites. The simulation details and the algorithm can be found in Supplementary Materials.

The calculation efficiency depends mainly on the polymer length and sampling of conformations. The following estimates were obtained for a machine equipped with NVIDIA Tesla V100 GPU and Intel Xeon Gold 6248 CPU. The calculation of the translocation time through a lipid membrane in the planar geometry, typical simulation time for a polymer chain of length $N=12$ is $0.0384 \pm 0.0004$ s per million of conformations and for a chain of length $N=16$ is $0.0784 \pm 0.0004$ s per million of conformations. This typical time allows for the generation of a significant statistical sampling for each pattern/sequence. For the spherical geometry of a core-shell micelle, the typical simulation time for the polymer of length $N=16$ is similar to planar geometry: $0.0834 \pm 0.0003$ s per million of conformations.

## Adaptive copolymer sequence

In this section we describe linear polymers which composition (sequence of monomers) is adapted to a given design criteria. Two examples are considered: the problem of optimization of passive translocation time through a lipid membrane represented by one dimensional energy barriers, and the problem of confined and constrained polymers on the example of the localization of a linear polymer in a concentric field of a spherical micelle.

### Translocating polymers

The problem of finding the composition of translocating polymer that minimizes the time of diffusive translocation through a lipid bilayer was studied in detail for homopolymers^30–32^ and amphiphilic copolymers^33^. Thus, we use these results as a reference to benchmark the predictions and the results of the enumeration-selection strategy.

A lipid membrane is represented by a one dimensional hydrophobic field of lipid tails $\Phi _{T}$ with a thickness of 6 lattice units, surrounded by a hydrophilic field of lipid heads $\Phi _{H}$ of two lattice units as shown in Fig. 3A. The rest of the box is filled by the solvent $\Phi _{S}$. We take for the measure of the design criteria the translocation time of a polymer with given sequence through the lipid membrane. The mean first escape time, $\tau$,

4 $$\begin{aligned} \begin{aligned} \tau \propto \int _{z_-}^{z^+}dz e^{\mathscr {F}\left( z\right) / k_{B}T}\int _{z_-}^{z}dz'e^{-\mathscr {F} \left( z'\right) / k_{B}T}, \end{aligned} \end{aligned}$$

is defined between $z_-$ and $z_+$ designating the position of the top and the bottom of the simulation box, correspondingly. Here, $\mathscr {F}(z)$ is the free energy of a layer *z* of conformations $\Gamma$, which have the center of mass, $\bar{z}(\Gamma )$, lying at a distance *z* from the bilayer’s mid-plane:

5 $$\begin{aligned} \begin{aligned} \mathscr {F}(z)=-k_BT\ln \sum _{\begin{array}{c} \Gamma ~\mathrm {with}\\ |\bar{z}-z|<\frac{1}{2}\end{array} }w_\Gamma e^{-\mathscr {H}(\Gamma )} \end{aligned} \end{aligned}$$

Here, $w_\Gamma$ is the bias of the distribution (in case of Rosenbluth generation it is a Rosenbluth weight^34^), and $\mathscr {H}(\Gamma )$ is the Hamiltonian of a configuration, Eq. (3). In the following, the lattice constant $1$ is used. It is important to note, that the polymer length-dependent diffusion constant is not considered in the expression (4) and thus, the following results represent timescales with respect to the self-diffusion time.

For simplicity of understanding the method, we assume that the monomers in a linear polymer chain can be of two types: hydrophobic ($P_T$, black) and hydrophilic ($P_H$, white). More types of monomers, branching of polymers and even gradual change between hydrophobic and hydrophilic monomers are possible extensions of the model. Each polymer sequence or pattern is thus a sequence of monomers of two kinds. The polymer properties are expected to be invariant under reversion of the sequence between both ends.

**Figure 5:**
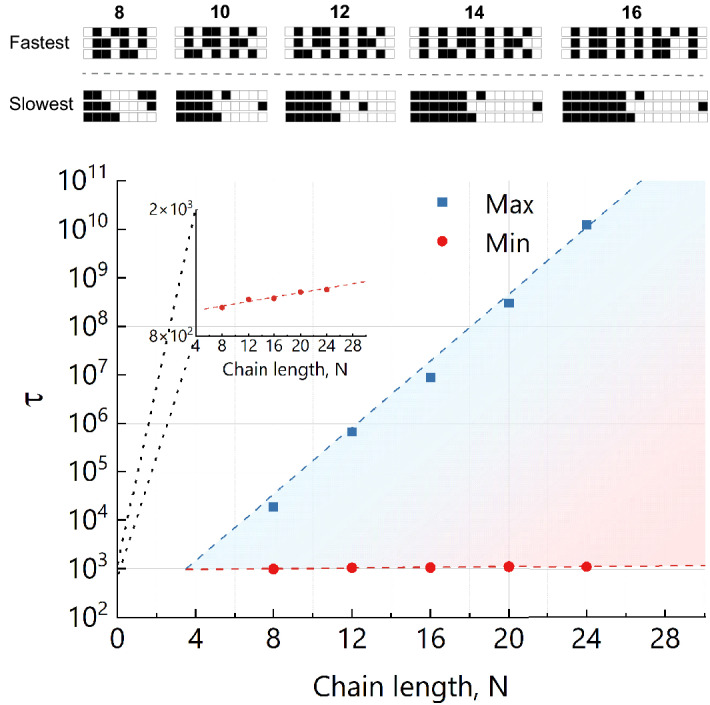
(**A**) Predicted fastest patterns (Min) and slowest patterns (Max), according to the mean escape time $\tau$ for a *fixed ratio* of hydrophobic (black) and hydrophilic (white) monomers, $N_{T}/(N_{T}+N_{H})=1/2$. Shown up to length $N=16$, while similar alternating patterns are observed for longer polymers (up to $N=24$, not shown for clarity). (**B**) The corresponding mean escape time $\tau$ of the slowest and fastest patterns as a function of the polymer length *N* for a fixed ratio, $N_{T}/(N_{T}+N_{H})=1/2$.

At a given ratio between hydrophobic and hydrophilic monomers, $N_{T}/(N_{T}+N_{H})$, the total number of possible sequences is $N!/({N_{T}!N_{H}!)}$, where $N=N_{T}+N_{H}$. It grows exponentially with the length of the polymer *N*. For each polymer sequence the exhaustive search method consists of the sampling of $~10^7$ independent self avoiding random walks that are uniformly distributed in the box and thus, samples sufficiently precise the free energy $\mathscr {F}$ (Eq. (4)) of conformations at each position *z*. Technically, the conformations are not stored in memory, but are only used to sample the free energy in parallel on GPU on-the-fly such that billions of conformations can be generated in a reasonable time.

During the generation process, the mean escape time $\tau$ is calculated taking into account all generated conformations. The process is performed for each sequence and each polymer length up to $N=24$. As a result, sequences can be ranked with respect to the time $\tau$.

The results for polymer length $N=12$ are presented in Fig. 4 and in Table S1. It shows that the translocation time is very sensitive to the sequence of the polymer. The most efficient translocation is found for the balanced hydrophobicity, $N_{T}/N=6/12$. This is consistent with the previous systematic findings using Monte Carlo simulations^30,31^. The difference of the mean escape time between different sequences is several orders of magnitude. At the balanced point, sequences with shortest hydrophilic/hydrophobic blocks show smallest translocation times only due to weak localization at the bilayer-solvent interfaces as compared to sequences with larger blocks. In accordance with a recent work^35^, we also see in Fig. 4 for slightly hydrophilic sequences, $N_{T}/N<6/12$, that formation of larger hydrophobic blocks near the center of the sequence leads to the smallest translocation times in this case.

Mean escape times show equivalent behavior as a function of the sequence for the various chain lengths investigated as shown in Fig. 5. There, the fraction of hydrophobic units is fixed to 1/2 for various polymer lengths. The results demonstrate that the sequences with alternating hydrophobic and hydrophilic monomers provide the fastest translocation time at balanced hydrophobicity. Both maximum and minimum data for $\tau (N)$ corresponding to alternating and diblock sequences can be approximated by an exponential increase with *N* (Fig. 5b). While there is only marginal increase of translocation time for alternating sequences which only weakly interact with the given diffusion barrier, for the slowest sequences (diblock copolymers) the slope is significantly larger, see Fig. 5b and Table S2. Assuming that the rate limiting process is the detachment of the hydrophobic block into solvent, the contact energy contribution is written as $z_{eff}\epsilon _{P_T-S} (N/2)$ with $z_{eff}$ being an effective coordination number counting the number of lattice contacts of $P_T$ monomers with solvent. The ratio $\sim 10^{7}$ between the maximum translocation time for $N=24$ (Fig. 5a), and the corresponding free diffusion result $\tau \sim |2z_\pm |^2\sim 10^3$ reflects an excess free energy barrier of $F_{barrier}=18k_BT$ for desorption of the diblock. An effective coordination number is then estimated as $z_{eff}\approx 15$, which is slightly smaller as compared to the result $z_{eff}\approx 19.6$ given in Ref.^35^. There, also contributions from conformational entropy change upon desorption as well as much shorter chains have been considered only. A more precise consideration will also take into account conformational entropy of confinement of the $P_T$-block into a slit-geometry given by the membrane thickness.

### Constrained and confined polymers

In this example, we address the problem of finding an optimal polymer sequence that can fit the best to a core-shell structure. It can be a problem of a maximum load of a block-copolymer micelle with a polymer or a packing problem of a polymer in a field of concentric layers. More generally, this can be an inverse problem of finding the molecular structure of the compound that creates a given field. In the following, we describe an example of inverse localization problem, *i.e.* a problem of finding a linear polymer sequence that is at a given position in space.

**Figure 6:**
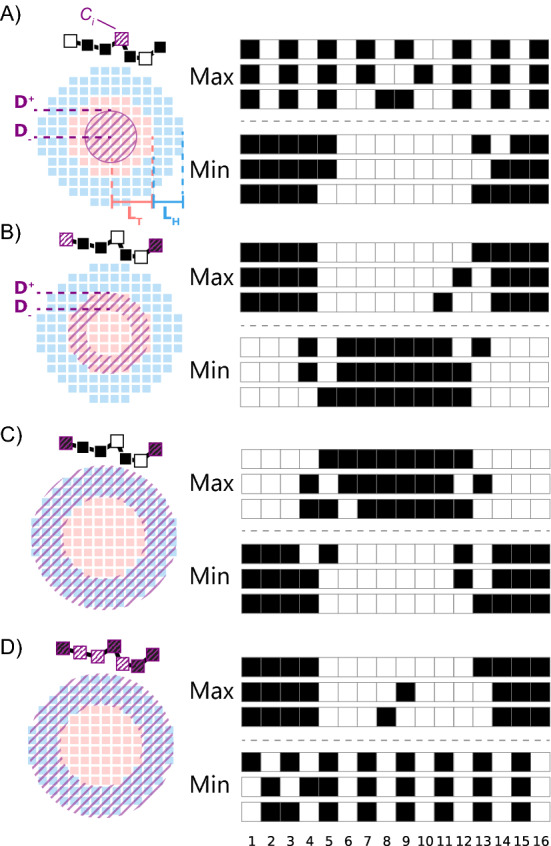
The localization problem of the given (purple) monomers in two concentric layers: hydrophobic core (red) and hydrophilic corona (blue). On the left: a schematic illustration of the criteria, on the right: three most probable and three less probable sequences obeying the criteria. Black is hydrophobic and white is hydrophilic monomers. (**A**) Central monomer $i=8$ should be localized in the center of the micelle, $0<r<2$, boundaries sketched as D_-_ and D_+_; (**B**) polymer ends $i=1$ and $i=16$ should be localized at the border between the core and the corona, $10<r<13$; (**C**) Polymer ends $i=1$ and $i=16$ should be localized in the corona, $13<r<16$; (**D**) All monomers $i=1, \dots ,16$ should be localized in the corona $13<r<16$.

We consider spherical symmetry in such a way that a given field represents concentric core-shell layers and we impose that a given monomer in the chain (hydrophilic or hydrophobic) has a maximum concentration in the chosen layer. To formulate this criteria for the localization problem, we define average concentrations of the i-monomer $\bar{C}_i(x,y,z)$ at a given position in space $C_i(x,y,z)$ as

6 $$\begin{aligned} \bar{C}_i(x,y,z)=\frac{1}{Z}\sum _\Gamma w_\Gamma e^{-H(\Gamma )} C_i(\Gamma ,x,y,z) \end{aligned}$$

where $\mathscr {H}(\Gamma )$ is the effective Hamiltonian, defined in Eq. (3), *Z* is the normalization constant, Eq. (2), and $C_i(x,y,z)$ is the concentration of the i-monomer of a conformation $\Gamma$ at a given point on space (*x*, *y*, *z*).

With a radial symmetry, (*x*, *y*, *z*) is reduced to concentric layer of radius *r* and the layer thickness $\sigma$. Additionally, we can fix the number of monomers $M\le N$ to be located in the concentric layer. The design criteria for the localization of i-monomer in the concentric layer $D=(r,r+\delta )$ can thus be formulated as the maximum of the fitting function $\theta _{MD}$

7 $$\begin{aligned} \begin{aligned} \theta _{MD}\propto \sum _{i=1}^{M}\sum ^{}_{r\in D} \bar{C}_i(r) \end{aligned} \end{aligned}$$

To illustrate this concept, we consider a concrete example of the micelle given by two concentric fields, hydrophobic in the core and hydrophilic in the corona. We fix the concentric field since we impose a polymer composition to adjust to that field. The field is defined as a hydrophobic layer with the radius $L_{T} = 13$ lattice units in the center and the hydrophilic shell of thickness $L_{H} = 3$ lattice units. The composition of amphiphilic copolymer of length $N=16$ with equal ratio between hydrophobic and hydrophilic monomers (hydrophobicity 50%), have to be adjusted to the given field in different variations, Fig. 6.

For such a polymer, the total number of sequence combinations is 12870. The predicted sequences that obey the localization criteria are sorted according to $\theta _{MD}$. Imposing different monomer localization criteria, the resulting sequence pattern can be very different. A) A central monomer $i=8$ of the sequence *M* (purple) should be localized in the core of the core-shell structure $(0<r<2)$. The best sequences correspond to strictly alternating copolymers, where the polymer passes through the core, thus allowing the central monomer to be located in the middle. Tri-block copolymers with hydrophobic ends are the worse rated according to this criteria. B) Monomers on both ends ($i=1$ and $i=16$) should be localized at the the border between hydrophobic and hydrophilic regions $(10<r<13)$ of a micelle. In this case the most adapted sequence is the the triblock polymer with two hyrdophilic ends and hydrophobic block in the middle. C) The ends should be located in the hydrophilic region $(13<r<16)$ of the micelle. A tri-block copolymer with hydrophilic ends is the most adapted sequence. D) The whole polymer should be located in the hydrophilic region $(13<r<16)$. Tri-block copolymer with a amphiphilic block in the middle and two hydrophobic ends are the most adapted sequences, while alternating copolymer is the less adapted.

## Reversible links and adaptive topology

In this section we introduce more complex molecular structures such as reversible links between molecules and hierarchical branching of molecules.

The increased complexity of the topology of the molecules allows for more degrees of freedom for the molecular design and provides a flexibility for adapting the molecule structure and composition to the changes of the environment in the accordance with the concept of dynamic combinatorial libraries for supra-molecular chemistry^36^. The range of complex structures that can be obtained with this strategy have received the name “dynamers”^37,38^ and some of them are depicted in Fig. 1. For example, reversible and dynamic links can be formed by hydrogen bonds or by recognition-directed reversible polyassociation through complementary interaction groups or reversible polycondensation and reversible functional groups. Classical example of a simple reversible system is a linear living polymer^39^. These various types of the reversibility can be implemented in a mean field through effective interactions between monomers and the solvent, while the average composition, length distributions, *etc*, are determined by the thermodynamic equilibrium. Shifting the equilibrium by changing external conditions leads to selectivity and responsiveness of a reversible self-assembled system.

### Hydrogen bonding

One of the form of reversible links is hydrogen bonds either between components or between monomers and water molecules. One of well-known examples is a polyethylene oxide (PEO) in water, which is water-soluble due to the formation of dynamic hydrogen bonds with water^40–42^. Hydrogen bonding can be described within a two-state model, where a monomer can be in two states: a monomer forming a hydrogen bond with water and a monomer without a hydrogen bond^43^. These monomers have different interaction parameters with water, while their position and number are determined from chemical equilibrium. The origin of two states is the gauche-trans equilibrium of monomer conformations that have different probabilities to form a hydrogen bond with water. Higher polymer concentration makes it more difficult to form a hydrogen bond, thus the probability to be in one or another state is concentration dependent.

**Figure 7:**
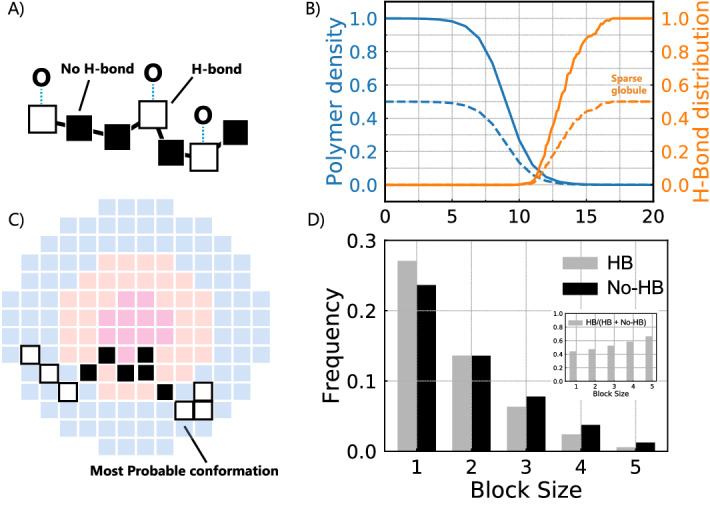
Dynamic formation of hydrogen bonds between monomers and water of a linear PEO chain. (**A**) A model of a two-state polymer, where black monomers correspond to the formation of a hydrogen bond with wate and white monomers correspond to no bond. The probability to have a bond is coupled with the polymer concentration. (**B**) Most probable configuration in the field of a polymer concentration. (**C**) Polymer concentration and the fraction of hydrogen bonds (orange) versus the distance from the center *r* of a two-state polymer in the polymer concentration field (blue). Dashed lines correspond to a sparse globule; (**D**) Distribution of block sizes blocks in the concentration field and their relative frequency, $f=f_{HB}/(f_{HB}+f_{no-HB})$. The corresponding H-bond distribution is proportional to solvent volume fraction.

As an example, consider a linear polymer consisting of $N=16$ monomers, where white monomers correspond to hydrogen bonding (hydrophilic) and black blocks correspond to free monomers (hydrophobic). The fraction of blocks is fixed to 50% and controlled by external conditions, but the position is not fixed and is coupled with the polymer concentration: $\phi _p=1.0 - 1.0/(1.0+\exp (-r+9.0))$ corresponds to a dense globule, solid line in Fig. 7C and $\phi _p=0.5 - 0.5/(1.0+\exp (-r+9.0))$ for a sparse globule, dashed line. In turn, the conformation with exact sequence of white and black blocks, depends on the distribution of the hydrophilic and hydrophobic blocks along the chain, Fig. 7. Running the search for a composition of a polymer in its own concentration field in a radial symmetry leads to the following results: the fraction of hydrogen bonds increases as the polymer density decreases with the distance from the center. If the density gradient of a polymer concentration is shifted, for example, due to changing external conditions such as temperature or pH, the fraction of hydrogen bonds would adjust to the field and the hydrogen bonds would re-distribute to adapt to the field.

### Hierarchical branching

**Figure 8:**
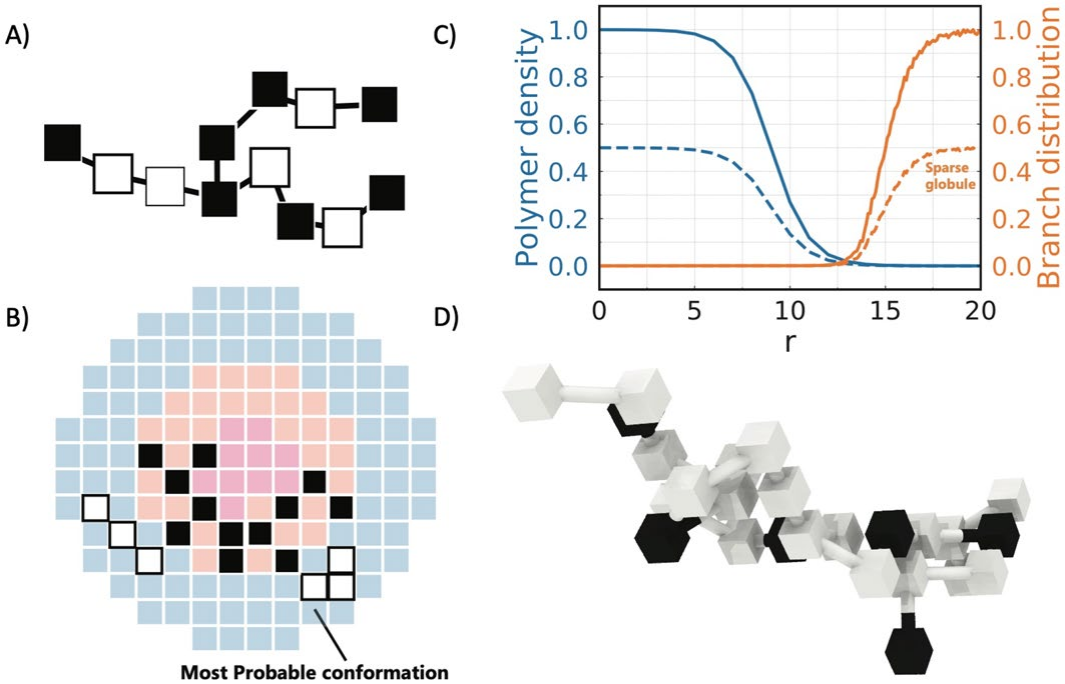
Adaptive branching of the molecules in the concentration field. (**A**) A model of a branched polymer, where black cubes designate hydrophobic monomers and white cubes designate hydrophilic monomers. The branching point is designated with two bonds to branches. (**B**) Most probable configuration of the branching polymer with a given probability of branching coupled with the field of polymer concentration. (**C**) Polymer concentration (blue) and the distribution of branching points of an adaptive polymer as a distance from the center *r*, with a given probability of branching $\theta =0.2$. Dashed lines correspond to a sparse globule. (**D**) Example of a 3D structure of a branched polymer conformation used in the study.

Often molecules are not linear and can have a complex topology such as branches or side groups. Branching provides an additional degree of freedom for the design of adaptive polymers. Considering the probability of branching $\theta$ for each generated monomer during Rosenbluth generation, different branching topologies can be obtained, Fig. 8. With this, the total number of branching points and the functionality of branches (number of branches at the branching point) can be controlled through external fields.

Hierarchical branching in Rosenbluth sampling is introduced as follows: the first monomer is placed at a random position in the simulation box. Next monomers are added one after another following a classic Rosenbluth chain growth algorithm and checking their self-avoidance. Starting from the third monomer, the polymer can branch with a given probability of branching $\theta$ which is checked according usual Monte Carlo procedure. The branching functionality *f* is introduced as the number of branches per branching point. Each branch is then followed in a recursive way and the random chain growth process continue until it reaches the given total polymer length. The resulting polymer provides the distribution of branches that follows the total density distribution, Fig. 8.

Such an approach allows to create molecules with adaptive topology that is controlled by external conditions. Examples could be polyimines^37,38^ derived from various diamines and dialdehydes that are sensitive to changes in absorption and fluorescence spectra as well as in their solubility in organic and aqueous media.

## Hybrid methods

Exact enumeration-selection computational strategy is a powerful tool for relatively small sets of conformation space. If the number of the building blocks increases, the number of conformations increases rapidly with the length *N* and generation of all conformations becomes impractical. One of the strategy is to use *representative* sampling, *i.e.* large enough sampling to ensure that the averages statistically do not depend on sampling, instead of a full sampling. This is also the solution for off-lattice models, where the method is readily generalized the same way as a standard SCMF theory with a representative sampling^27^. Further, the enumeration method can be combined and complemented with heuristic methods for efficient search in the parameter space such as neural networks (NNs), genetic algorithms or any other method for exploration in large parameter space. This combination still relies on large conformational sampling to properly sample the statistical conformations of a single chain.

To illustrate such coupling and complementarity of the methods, a special neural network was designed to learn the relation between the structure (block copolymer sequence) and the translocation time through the membrane (target fitting function) with the same model parameters as we used for exact enumeration for the problem of finding a polymer sequence for optimal translocation through the membrane (first example). The method and the structure of the network is described in Ref.^35^.

The comparison allows to reveal the fundamental differences in the approaches and highlight the conceptual strengths and weaknesses of both approaches that can be combined. The enumeration-selection strategy implies direct exploration of the whole conformational and parameter space. This gives exact answer what is the optimal composition without the risk to be trapped in subset of parameters corresponding to a local minimum. In turn, NN provides the prediction for optimal solution based on training set and entirely depends on how large and complete this set is. Machine learning does not guarantee that the predicted solution is the true optimal solution corresponding to the global minimum, thus often called a black box without possibility to verify the predicted results. However, NNs are generally efficient in exploration of large sets of parameters, while the time of computation for achieving the optimal solution with enumeration strategy increases exponentially with the size of the system and number of parameters to explore, limiting its applicability. Thus, a combination of machine learning with exact enumeration-selection strategy with a balanced sampling generated by exact enumeration for training of NN could be the best strategy for solving design problems.

**Figure 9:**
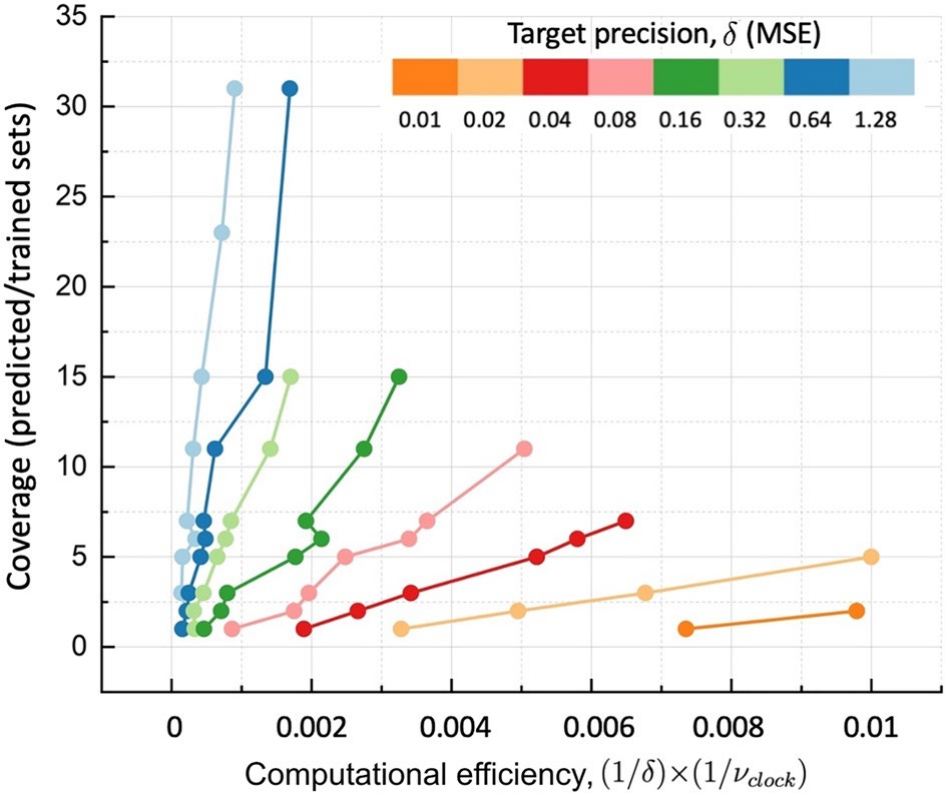
Evaluation of the performance of the hybrid prediction method, based on convolutional neural network (CNN) with exact enumeration used as a training set. $\delta$ is the mean square error (target precision) and $\nu _{clock}$ is the number of processor clock cycles multiplied by the number of parallel threads employed in units of 10^12^ cycles.

The comparison between the performances of enumeration-selection and neural network methods is demonstrated on the example of a sequence of an amphiphilic polymer of length $N=14$ monomers. In particular, the performance of NN depends on the size of the training set. This training set (training window) is generated by exact enumeration for which the result is known exactly. The prediction of NN is evaluated on a predicted set, while the performance of NN is defined as a ratio between predicted and training sets. NN can correctly predict much larger sets for the price of precision. In this example, the targeted precision for translocation time $\tau$ is given as a mean squared error (MSE) $\delta$ of the fitting function $\log \tau$, while the computational efficiency is measured as $(1/\delta )\times (1/\nu _{clock})$, where $\nu _{clock}$ is the total number of (parallel) clock cycles (in Tera-clocks) spent for the prediction, Fig. 9. The performance is evaluated as the fraction between predicted sequences to sequences used for training (sampling set obtained by exact enumeration).

Three conclusions can be made from the comparison with NN method: (i) A high precision of machine learning prediction can only be achieved by a large training set, which can be generated by exact enumeration, and as a counterexample, the yellow curve is larger than the red or pink or green(s) curves in Fig. 9. (ii) For a given precision, the NN reduces the computational effort most for the higher precision. (the slope of the orange curve is smaller than that of the blue). (iii) Computational efficiency of a hybrid method, which includes exact enumeration of the training set and NN for prediction for “unseen” conformations set, relies on the size of the training set. For any given precision, a higher fraction of NN contribution reduces the computational cost.

In brief, the exact enumeration can be used as a ground truth for creation of the training set for training the neural network, while machine learning can extend the method for exploration of large systems and large parameter space (heuristic exploration), thus providing a powerful combination of inter-related complementary methods.

## Conclusions

We presented exhaustive search method for design of composition of polymer sequences in the mean field approximation. This method allows to solve inverse (or design) problem with brute-force solution of direct problem. The key of performance is massive parallelization on GPUs which almost eliminates communication between cores which makes this method significantly more efficient than existing computationally expensive heuristic optimization methods such as deep learning, neural networks or genetic algorithms. The performance of the method was demonstrated on two examples: polymer sequence that translocate through lipid bilayer with a minimal time and the the sequence with best affinity to spherical core-shell structure, representing a micelle. This method is readily generalized to wide spectra of inverse (or design) and combinatorial search problems in chemistry and physics.

## Supplementary Information

Supplementary Information.

## Data Availability

All data generated or analyzed during this study are included in this published article and its supplementary information files.
