# Supplementary material for: Efficient enumeration-selection computational strategy for adaptive chemistry
Source: Sci Rep. 2022 Aug 29;12:14664. doi: 10.1038/s41598-022-17938-x (PMC9424279; doi:10.1038/s41598-022-17938-x)
Supplement: Supplementary file 1 — Supplementary Information. [file 41598_2022_17938_MOESM1_ESM.pdf]

Efficient enumeration-selection computational strategy for adaptive chemistry
Supplementary Materials

Yachong Guo

Kuang Yaming Honors School, Nanjing University, Nanjing 210023, China

Marco Werner

Leibniz-Institut für Polymerforschung Dresden e.V.,

Hohe Straße 6, 01069 Dresden, Germany

Vladimir A. Baulin*

Departament d'Enginyeria Química,

Universitat Rovira i Virgili 26 Av. dels Paisos Catalans, 43007 Tarragona, Spain

(Dated: May 31, 2022)

COMPUTATIONAL DETAILS OF THE ENUMERATION-SELECTION STRATEGY

The implemented enumeration-selection computational strategy for inverse design problems is based on efficient parallelization scheme on Graphics Processing Units (GPUs). This strategy allows to perform independent Monte Carlo simulations of a conformation of a single polymer chain of arbitrary topology on each GPU core using C++ and CUDA library. The realization has a modular structure which makes it very flexible for modification, further development and extension for new applications. The implementation is divided in 3 modules:

1. First module is the controlling module. It controls core process such as the I/O data flow between host and device, optimization method and selection strategy to explore the polymer properties: specific max/min objectives, choose of the optimization method, such as genetic algorithms. This module specifies the geometry and discretization of space taking into account the symmetry of the system: lattice parameters, grid dimensions, symmetry of the system (cylindrical, spherical, planar or 3D) and the size of the box.

2. The second module defines force fields and topology of molecules, which is determined by the range of parameters: monomer composition, branching, annealing. In this realization, $N * M$ dimensional array is allocated in the host memory then transferred to the global memory for the Hamiltonian calculation, where N is the total number of monomers in a single molecule (in case it is fixed and not a design parameter) and M is the number of topology parameters associated with the structure of the molecule. More parameters are related to the structure of the molecule and polymer topology: linear or branched polymer, annealed or fixed ration of hydrophobic and hydrophilic blocks, interaction strength and range. As a result, molecule structure is represented by $(3 + N)$-dimensional array stored in the constant memory, where N is the number of units or beads composing a polymer molecule (polymer length). For a cubic lattice, it contains the description of the geometry and division of the simulation box into set of auxiliary cells.

3. The third module is the sampling generator, which generates the molecule conformations and calculates the interactions according to the Eq 3 of the main text. The code for the generation of molecules according to a given statistical distribution can be changed independently from other parts of the program. In our realization, the chains are gener-

ated according to GPU parallel version of the Rosenbluth algorithm, Ref. 29 of the main text, providing uncorrelated self-avoiding walks, which can run on each CUDA thread independently, namely, the full computational task of parallel random monomer selection and random self-avoiding walks can be programmed at once in a single GPU kernel. Thus, each polymer conformation is generated with each CUDA thread (Algorithm 1).

In this Algorithm the number of blocks $NumBlock$ is defined as the total number of molecules to be generated divided by the number of threads per block. In this way each confirmation in each block is tagged by the thread number while generated. All the coordinates and weights associated with each conformation are stored in the shared memory. The first monomer is placed at the center of the coordinate system or it can also be placed at any position in the simulation box depending on realization. The second bond vector is randomly chosen within all $z = 26$ possible lattice space in 3D space and added to the monomer. Starting from the third monomer, a bias is introduced to the position of a new monomer because the new monomer may overlap with the previous monomers. This is taken into consideration in the so-called Rosenbluth weight, which is the probability of positioning a new monomer avoiding overlaps with previous beads.

For the 3D geometry of cubic lattice, the Rosenbluth weight is cumulatively calculated after the whole chain is generated. During the chain generation, next monomer position is randomly chosen from the $z = 26$ neighbors of the present bead. This position is accepted if there is no overlap with all previously generated beads.

The random chain growth process will be continued if the chain is self-avoiding. However, if the chain grow to a dead end when all possible nearby cells are occupied by previous monomers, the whole generated sequence, obtained up to this point, must be discarded, and the process starts from the first step again. Repeating these steps a conformation of a self-avoiding polymer molecule of a given length N with a calculated Rosenbluth bias is the result of this simulation of each CUDA thread. Since the length N is the same for all statistical conformations, the threads will finish calculation at roughly the same time.

The interaction with constant fields is also calculated during the chain generation using Eq. 3. Once the chain generation is finished, the total partition function of the system is also obtained from the modules. With this, it gives the access to all the thermodynamic properties of the system.

Once all conformations of the chain are generated, the terminal signal is received by

Algorithm 1 Lattice Rosenbluth chain generation

```
Require: BlockDim.x is the block dimension defined by user
Require: seed is a random number seed chosen by the user
Require: chainlength is the polymer chain length chosen by the user
Require: Function rngonlattice generates a random position for the next monomer
Require: Function distance calculates the distance between two monomers
x, y, z ← BlockDim.x ∗ chainlength
w ← BlockDim.x
rng ← rng(seed)
function ChainGeneration
    for i = 0 → chainlength do
        if i == 1 then
            Pos[0] ← 0                    ▷ Pos[i] denotes position of monomer i (x_i, y_i, z_i)
            p ← 1/6                       ▷ p denotes Rosenbluth weight
        end if
        for k = 0 → ptMax do              ▷ ptMax denotes total trial attempts
            Pos[Temp] ← rngonlattice
            Pos[Temp] = Pos[i − 1] + Pos[Temp]
            overlap ← False
            for j = 0 → i − 1 do
                if Pos[j] ∧ Pos[Temp] then
                    overlap ← True
                end if
            end for
            if ¬overlap then
                Pos[i] = Pos[Temp]
                break
            end if
        end for
    end for
    for i = 1 → chainlength − 1 do
        KK ← 0
        for j = 0 → i − 1 do
            R2 ← distance(Pos[i], Pos[j])
            if R2 ≤ d_max then            ▷ d_max denotes the maximum polymer bondlength
                KK = KK + 1
            end if
        end for
        p = p ∗ (1.0/(Cod − kk))
    end for
end function
```

the control module and the selection of the optimal sequence can be performed before the generation of the sampling of another sequence.

STATISTICAL ANALYSIS OF A LINEAR POLYMER SEQUENCE

The statistical analysis of the pattern of a linear chain of fixed length $N = 12$ and variable ratio of hydrophobic and hydrophilic monomers, H/T is shown in Table S1. We studied statistical distribution of the average number of blocks, calculated by counting all H and T blocks and averaging 10 sequences of each group. Another characteristics is the average blocks sizes of H and T, calculated as the total block size divided by the total number of blocks. Both average number of blocks and blocks sizes were calculated from separated two groups: sequences with 10 maximum and 10 minimum values of τ.

There is a significant difference in the number of blocks and the block sizes between the fastest and slowest patterns. The number of blocks of the fastest patterns is much larger than that of the slowest patterns and there is an opposite trend for the block size. Both the number of blocks and the block size change monotonously with H/T ratio. The fastest patterns have a balanced distribution of hydrophobic and hydrophilic blocks with the ratio $H/T \sim 50\%$ and the average block size is close to minimal possible, *i.e.* 1 monomer.

Statistical analysis of the pattern of the sequences with a balanced ratio H/T=50% is shown in Table S2. Three fastest and three slowest patterns corresponding to a minimum and a maximum of the mean escape time τ of a linear polymer chain with a fixed H/T ratio are shown in Fig. 5 of the main text. The results demonstrate that the fastest patterns consist of large number of alternating hydrophobic and hydrophilic blocks, which are not regular with few blocks grouped together. The slowest blocks resemble di-block copolymers, which are also not regular.

The fastest patterns (Min group) exhibit a positive correlation between the number of hydrophobic monomers T and the average number of blocks N_T. The T block size S_T is relatively small, ranging between 1.06 and 1.30, with a tendency of increase with the total length of the polymer. The block size of H, N_H is slightly bigger than N_T and is ranging between 1.20 and 1.36. The slowest patterns (Max group) demonstrate the average number of blocks equal to 1.67 with an average of 3 total blocks. Thus, the translocation behavior is strongly affected by the block size. The block size tends to stay as small as possible to allow for fast translocation, and correspondingly, the number of blocks is relatively high, thus suggesting a design solution in form of an alternating co-polymer with smallest block size.

Table S1. Statistical analysis of a linear polymer chain with fixed length $N = 12$ and variable ratio of hydrophilic/hydrophobic blocks

T	Ra	Min				Max			
		N_T	S_T	N_H	S_H	N_T	S_T	N_H	S_H
0	0%	0.00	0.00	1.00	12.00	0.00	0.00	1.00	12.00
1	8%	1.00	1.00	2.00	5.50	0.83	1.00	1.50	7.22
2	17%	1.33	1.50	2.33	4.29	1.67	1.00	1.83	5.18
3	25%	1.33	2.25	2.33	3.86	2.00	1.33	1.67	5.20
4	33%	1.50	2.67	2.50	3.20	1.50	2.56	1.17	6.71
5	42%	2.00	2.50	3.00	2.33	1.50	3.22	1.33	5.25
6	50%	5.17	1.13	5.17	1.00	1.67	3.60	1.67	3.60
7	58%	4.67	1.36	4.67	1.00	1.67	4.20	1.67	3.00
8	67%	4.00	1.79	4.00	1.00	1.50	5.22	1.33	3.00
9	75%	3.33	2.50	3.00	1.00	1.50	5.89	1.33	2.25
10	83%	2.67	3.63	2.00	1.00	1.50	6.56	1.17	1.57
11	92%	1.83	5.91	1.00	1.00	1.67	6.60	0.83	1.00
12	100%	1.0	12.00	0.00	0.00	1.0	12.00	0.00	0.00

Note: T: Number of hydrophobic monomers in the chain

N: Polymer chain length (total number of monomers)

Ra: Hydrophobicity $Ra = (N_T/(N_H + N_T)))$

N_T: Average number of T blocks

S_T: Average size of block T

N_H: Average number of H blocks

S_H: Average size of block H

Table S2. Statistical analysis of a linear chain with variable lengths, $N = 8 - 16$ and fixed to 50% ratio of hydrophobic/hydrophilic blocks

T	N	Min				Max			
		N_T	S_T	N_H	S_H	N_T	S_T	N_H	S_H
4	8	3.00	1.06	3.17	1.21	1.67	2.40	1.67	2.40
5	10	3.67	1.09	3.67	1.36	1.67	3.00	1.67	3.00
6	12	4.67	1.07	5.00	1.20	1.67	3.60	1.67	3.60
7	14	5.33	1.22	5.00	1.30	1.67	4.20	1.67	4.20
8	16	5.50	1.30	6.00	1.31	1.67	4.80	1.67	4.80

Note: T: Number of hydrophobic monomers in the chain

N: Polymer chain length (total number of monomers)

N_T: Average number of T blocks

S_T: Average size of block T

N_H: Average number of H blocks

S_H: Average size of block H
